# Artificial Intelligence, Intrapartum Ultrasound and Dystocic Delivery: AIDA (Artificial Intelligence Dystocia Algorithm), a Promising Helping Decision Support System

**DOI:** 10.3390/jimaging10050107

**Published:** 2024-04-29

**Authors:** Antonio Malvasi, Lorenzo E. Malgieri, Ettore Cicinelli, Antonella Vimercati, Antonio D’Amato, Miriam Dellino, Giuseppe Trojano, Tommaso Difonzo, Renata Beck, Andrea Tinelli

**Affiliations:** 1 Department of Interdisciplinary Medicine (DIM), Unit of Obstetrics and Gynecology, University of Bari “Aldo Moro”, Policlinico of Bari, Piazza Giulio Cesare 11, 70124 Bari, Italy; antoniomalvasi@gmail.com (A.M.); ettore.cicinelli@uniba.it (E.C.); antoniodamato19@libero.it (A.D.); miriam.dellino@uniba.it (M.D.); difonzo.tommaso.md@gmail.com (T.D.); 2 FIAT-ENI, Environmental Companies, and Chief Innovation Officer in CLE, 70124 Bari, Italy; lorenzo@malgieri.org; 3 Department of Precision and Regenerative Medicine and Jonic Area, University of Bari “Aldo Moro”, 70121 Bari, Italy; antonella.vimercati@uniba.it; 4 Department of Maternal, Child Gynecologic Oncology Unit, “Madonna delle Grazie” Hospital ASM, 75100 Matera, Italy; giutrojano@gmail.com; 5 Department of Medical and Surgical Sciences, Anesthesia and Intensive Care Unit, Policlinico Riuniti Foggia, University of Foggia, 71122 Foggia, Italy; beckrenata64@gmail.com; 6 Department of Obstetrics and Gynecology and CERICSAL (CEntro di RIcerca Clinico SALentino), Veris delli Ponti Hospital Scorrano, 73020 Lecce, Italy

**Keywords:** artificial intelligence, intrapartum ultrasound, dystocia, asynclitism, labor, cesarean section, vaginal operative delivery, malrotation, malposition

## Abstract

The position of the fetal head during engagement and progression in the birth canal is the primary cause of dystocic labor and arrest of progression, often due to malposition and malrotation. The authors performed an investigation on pregnant women in labor, who all underwent vaginal digital examination by obstetricians and midwives as well as intrapartum ultrasonography to collect four “geometric parameters”, measured in all the women. All parameters were measured using artificial intelligence and machine learning algorithms, called AIDA (artificial intelligence dystocia algorithm), which incorporates a human-in-the-loop approach, that is, to use AI (artificial intelligence) algorithms that prioritize the physician’s decision and explainable artificial intelligence (XAI). The AIDA was structured into five classes. After a number of “geometric parameters” were collected, the data obtained from the AIDA analysis were entered into a red, yellow, or green zone, linked to the analysis of the progress of labor. Using the AIDA analysis, we were able to identify five reference classes for patients in labor, each of which had a certain sort of birth outcome. A 100% cesarean birth prediction was made in two of these five classes. The use of artificial intelligence, through the evaluation of certain obstetric parameters in specific decision-making algorithms, allows physicians to systematically understand how the results of the algorithms can be explained. This approach can be useful in evaluating the progress of labor and predicting the labor outcome, including spontaneous, whether operative VD (vaginal delivery) should be attempted, or if ICD (intrapartum cesarean delivery) is preferable or necessary.

## 1. Introduction

One of the hardest processes to overcome during labor is the positioning of the fetal head during engagement and progression in the birth canal. These malpositions and malrotations are also common causes of dystocic labor and arrest of progression, which necessitates a surgical delivery [1,2]. Traditionally, a vaginal digital examination is used to assess the fetal head position and engagement. The American College of Obstetricians and Gynecologists (ACOG) frequently describes the location of the fetal head in the pelvis with reference to an imagined horizontal plane at the level of the ischial spines [3]. The degree of fetal engagement and progression is delineated by different planes of the pelvis, which have values ranging from −5 (5 cm above the ischial spines) to +5 (5 cm below the ischial spines with the fetal head visible at the introitus). The head is considered engaged when the leading point of the skull touches the ischial spine plane; this is referred to as station 0.

This classification has been criticized as inaccurate and poorly reproducible, as reported by Dupuis et al. in a study evaluating the reliability of the transvaginal assessment of fetal head stations using a birth simulator [4]. The clinical ACOG classification may have a significant impact on the diagnosis of dystocia and delivery outcomes [5]. This may contribute to the failure of instrumental delivery, which may reach up to 10% [6]. Errors in the diagnosis of the fetal head station can have major implications during labor and adverse perinatal outcomes, such as acidemia, fetal traumas, intracranial hemorrhages, and low Apgar scores after an emergency cesarean section for failed operative vaginal deliveries. Many researchers have proposed many parameters to evaluate the engagement, descent, and internal rotation of the fetal head in the birth canal [7]. With the use of artificial intelligence (AI), we tried to evaluate some geometric parameters measured using an intrapartum ultrasound to understand whether the type of delivery outcome, ICD or non-ICD, can be predicted, building a specific algorithm.

## 2. Materials and Methods

The study was conducted in the period between January 2014 and December 2020 by collecting data from the Department of Obstetrics and Gynecology of the following Italian Hospitals: “Santa Maria” Hospital, “Vito Fazzi” Hospital, and “Veris Delli Ponti” Hospital.

This retrospective study was approved by the Institutional Review Board (CER 0320). The study was conducted in accordance with the Declaration of Helsinki, with data anonymization to prevent the patients from being recognized. We chose to carry out a retrospective study as we had all the labor delivery parameters useful for computer analysis and the construction of the data to be inserted into the database, therefore starting backward, from birth to labor. This study retrospectively analyzed the clinical data of only full-term nulliparous pregnant women with singleton fetuses with a cephalic presentation under neuraxial analgesia with intrapartum ultrasonography (IU) in the prolonged second stage of labor. The prolonged second stage of labor for nulliparous with neuraxial labor analgesia was defined, according to the ACOG, as more than three hours [8,9]. Patients’ exclusion criteria were fetuses in the breech, transverse, or oblique presentation, twin pregnancies, abnormal placental implantation, HELLP syndrome, coagulation disorders, a scarred uterus, non-reassuring FHR, thick meconium, and cephalopelvic disproportion. The patients agreed for their collected data to be used anonymously for the scientific purposes of building possible analysis software and decision-making algorithms by signing a specific form in a medical record. In this way, none of the patients could be recognizable in the collected data. All patients underwent vaginal digital examination by obstetricians and midwives during labor and delivery, and the evaluation of the visits was reported not only in the medical record but also inserted into specific databases for analysis. The age, gestational age, body mass index, neonatal weight, Apgar score at 1 min, Apgar score at 5 min, cephalic presentation (occiput anterior, posterior, or transverse), maternal complications, and outcome of delivery (intrapartum cesarean delivery, operative vaginal delivery, or spontaneous delivery) were anonymized and classified in tables. In addition, all patients underwent IU, and the following “four geometric parameters” were measured for all patients at three hours: (1) angle of progression (AoP), (2) asynclitism degree (AD), (3) fetal head–symphysis distance (HSD), and (4) midline angle (MLA). All four geometric parameters were researched and evaluated using normal ultrasound transabdominal probes of 3.5 MHz.

In daily practice, the physician’s decision to perform an ICD or not is also based on their experience and evidence. An algorithm, with the acronym AIDA (artificial intelligence dystocia algorithm), was built by us by foregrounding the following factors: (A) humans in the loop, that is, to use AI algorithms that prioritize the physician’s decision based on their experience; (B) explainable artificial intelligence (XAI), using an algorithm that allows the physician to systematically understand how the results of the algorithms can be explained and traced back to an explanation and understanding the phenomenon being investigated. 

The problem that the AIDA aims to answer is the following: knowing the values of the four geometric parameters and the delivery outcome, ICD or non-ICD, if it is possible to predict the delivery outcome for new cases, and how reliable the prediction will be. 

To answer this question, the AIDA was built into four steps. The first step uses Pearson’s correlation, which measures the linear correlation between two sets of data to verify that the four geometric parameters are uncorrelated or have non-significant correlations. The second step uses a series of supervised machine algorithms applied to the four geometric parameters and the physician-determined delivery outcome. The prediction performance of the algorithms is measured to choose at least three of the best-performing ones. The third step is to identify the cut-off values of the four geometric parameters associated with the ICD delivery outcome and those associated with the non-ICD delivery outcome. Following the calculation of the cut-off values, five AIDA classes are defined, depending on the number of parameters that are in the ICD zone or the non-ICD zone. The fourth step is to perform the delivery prediction based on the values of the four geometric parameters using the three best-performing algorithms identified in step two, with the relevant AIDA class described in step three allowing the physician to evaluate the clinical reliability of the prediction, i.e., ICD or non-ICD.

### 2.1. AIDA’s First Step

In the first step, a traditional statistical analysis method was used. The authors investigated the correlation between these parameters using Pearson’s correlation: Apgar scores at 1 min, Apgar scores at 5 min, Angle of progression (AoP), Asynclitism degree (AD), fetal head–symphysis distance (HSD), and midline angle (MLA). Pearson’s coefficient helped quantify the correlation, providing information about the magnitude of the association, as well as the direction of the relationship. Pearson’s coefficients range from +1 to −1, where +1 indicates a perfect positive monotonic relationship between the paired data, −1 indicates a perfect negative relationship, 0 indicates that no relationship exists; between ±0.00 and ±0.19 indicates a very weak degree, between ±0.20 and ±0.39 indicates a weak degree, between ±0.40 and ±0.59 indicates a moderate degree, between ±0.60 and ±0.79 indicates a strong degree, and between ±0.80 and ±1.00 indicates a very strong degree of correlation. The *p*-value helps assess whether a correlation is statistically significant, and a *p*-value < 0.05 * was considered statistically significant, using the asterisk system to denote significance (* *p* < 0.05 *, *p* < 0.01 **, *p* < 0.001 ***).

### 2.2. AIDA’s Second Step

In the second step, various machine learning algorithms were utilized, including random forest, XGBoost, logistic regression, decision tree, SVM (support vector machine), and MLP (multi-layer perceptron) to classify and predict the following delivery outcomes using the geometric parameters: intrapartum cesarean delivery (ICD) or non-cesarean delivery (operative vaginal delivery or spontaneous delivery). To evaluate the performance of each machine learning algorithm, the ROC (receiver operating characteristic curve), AUC (area under the ROC curve), accuracy, precision, recall or sensitivity, specificity, F1 score, and confusion matrix [10] were calculated. These metrics allowed for a more detailed analysis of the performance of the machine learning algorithms. In the confusion matrix, true positive (TP) represents the number of delivery outcomes that were ICD and classified as ICD, true negative (TN) represents the number of delivery outcomes that were non-ICD and classified as non-ICD, false positive (FP) represents the number of delivery outcomes that were non-ICD but classified as ICD, and false negative (FN) represents the delivery outcomes that were ICD but classified as non-ICD. The equation used to evaluate the accuracy was (TP + FP)/(TP + TN + FP + FN), where the total number of ICD-predicted ICD (TP) and non-ICD predicted non-ICD is divided by the total number of outcomes in the dataset (TP + TN + FP + FN); the highest possible value for the precision is 1.0 when the FP is zero. The equation used to evaluate the precision was (TP)/(TP + FP), where the total number of ICD-predicted ICD (TP) is divided by the total number of ICD outcomes in the dataset (TP + FP); the highest possible value for precision is 1.0 when the FP is zero. The equation used to evaluate recall or sensitivity was (TP)/(TP + FN), where the total number of ICD-predicted ICD (TP) divided the total number of ICD-predicted ICD (TP + FP); the highest possible value for recall or sensitivity is 1.0 when the FP is zero. The equation used to evaluate the specificity was (TN)/(TN + FP), where the total number of non-ICD predicted non-ICD (TP) is divided by the total number of non-ICD outcomes in the dataset; the highest possible value for specificity is 1.0 when the FP is zero. The F1 score is the harmonic mean of the precision and recall. The highest possible value for an F1 score is 1.0, indicating that either the precision or recall is 1.0, while the lowest possible value is zero when either the precision or recall is zero.

### 2.3. AIDA’s Third Step

In the third step, a decision tree (DT) was employed to identify the values of the four geometric parameters associated with the delivery outcomes of ICD and those associated with the delivery outcomes of non-ICD. Among the machine learning algorithms, the decision tree is the most easily understood and suitable for classifying something. The decision tree was applied to each pair of geometric parameters: AoP-AD, HSD-AD, MLA-AD, HSD-AoP, AoP-MLA, and HSD-MLA. For each geometric parameter, the cut-off value or range between the delivery outcomes of ICD and non-ICD was calculated. In our database, each value of the four geometric parameters was color-coded for visual and intellectual simplicity: green was used if the decision tree result classified it as belonging to the non-ICD delivery outcome, and red was used if the delivery outcome was classified as belonging to the ICD delivery outcome. The values of the four geometric parameters falling within a cut-off range were colored yellow.

The dataset was partitioned into five AIDA classes as follows: (a) AIDA class 0 included all patients who had all four green parameters; (b) AIDA class 1 included all patients who had one red or yellow parameter and three green parameters; (c) AIDA class 2 included all patients who had two red or yellow parameters and two green parameters; (d) AIDA class 3 included all patients who had three red or yellow parameters and one green parameter; (e) AIDA class 4 included all patients who had all four red or yellow parameters.

### 2.4. AIDA’s Fourth Step

In the fourth step, various machine learning algorithms were employed to predict the delivery outcome. In more detail, 70–30 separation was used, with 70% of the data allocated for training (95 patients), and the remaining 30% of the data (40 patients) was used for testing and validation. Each patient’s data record contained not only the delivery outcome but also the ID used to detect the patient’s AIDA class. The performance of each machine learning algorithm, as defined in the second level, was evaluated for all 40 patients and for each AIDA class individually.

The software used for AI analysis was AlterixDesigner with AlterixAI, V 2023.2.1.89, and IBM SPSS Statistics V 29.0.2.0 (20). In AlterixAI, the “create samples” tool was used to obtain five different 70–30 separation samples by setting the random seed parameter. This parameter, an integer value between 1 and 1000, determined which sample an individual row of data was placed in for the estimation (70%) and validation (30%) samples. The default value of 1 was the recommended choice, while additional values of 0, 250, 500, and 750 were also tested. Multi-layer perceptron (MLP), a neural network with one hidden layer, was included in IBM SPSS, while all other algorithms were implemented in AlterixDesigner.

## 3. Results

The authors examined a total of 135 patients involved in the study, with the following demographics: (a) age (years): mean 31.62 and standard deviation: 5.28; (b) gestational age (weeks): mean: 40.16 and standard deviation: 1.02; (c) gestational age (days): mean: 283.09 and standard deviation: 7.15; (d) BMI: mean: 27.52 and standard deviation: 2.95; (e) neonatal weight (grams): mean: 3926.68 and standard deviation: 309.66; (f) Apgar score (1 min): mean: 6.65 and standard deviation: 1.22; (g) Apgar score (5 min): mean 8.74 and standard deviation: 1.12.

The descriptive statistics for all four geometric parameters of the 135 patients involved in the study were as follows (minimum/maximum/mean/standard deviation): (1) head–symphysis distance (HSD) (mm): min: 10, max: 51, mean: 21.47, standard deviation: 9.265; (2) asynclitism degree (AD): (mm): min: 4, max: 95, mean: 60.18, standard deviation: 18.866; (3) midline angle (MLA) (°): min: 26, max: 90, mean: 62.59, standard deviation: 14.986; (4) angle of progression (AoP) (°): min: 72, max: 192, mean: 122.75, standard deviation: 27.454.

### 3.1. Findings from the AIDA’s First Step: Pearson’s Correlations

Pearson’s correlation between the Apgar scores at 1 min and 5 min showed a very strong and statistically significant correlation between them (*PC* = 0.8, *p*-value < 0.001). This aspect also demonstrated the genesis of the two quantities, which are the consequence of the same evaluation carried out 5 min apart from each other and, therefore, are necessarily linked to each other.

The correlations between the four geometric parameters were: (a) AoP-AD, weak and statistically significant (*PC* = 0.36, *p*-value < 0.001), (b) AoP-HSD, very weak and non-statistically significant (*PC* = −0.12), (c) AoP-MLA, weak and statistically significant (*PC* = −0.27, *p*-value < 0.001), (d) HSD-AD, very weak and statistically significant (*PC* = 0.18, *p*-value < 0.05), (e) AD-MLA, very weak and non-statistically significant (*PC* = 0.14), and (f) HSD-MLA, moderate and statistically significant (*PC* = 0.36, *p*-value < 0.001).

The correlation between the Apgar scores at 1 min and the geometric parameters was statistically significant and very weak with AoP (*PC* = 0.19, *p*-value < 0.001), weak with AD (*PC* = −0.2, *p*-value < 0.05) and MLA (*PC* = −0.31, *p*-value < 0.001), and very weak and non-statistically significant with HSD (*PC* = −0.12).

The correlation between the Apgar scores at 5 min and the geometric parameters were statistically significant and very weak with AoP (*PC* = 0.18, *p*-value < 0.05) and AD (*PC* = −0.19, *p*-value < 0.05), weak with MLA (*PC* = −0.24, *p*-value < 0.01), and small and non-statistically significant with HSD (*PC* = −0.12).

All these data are reported in Table 1.

**Figure 1:**
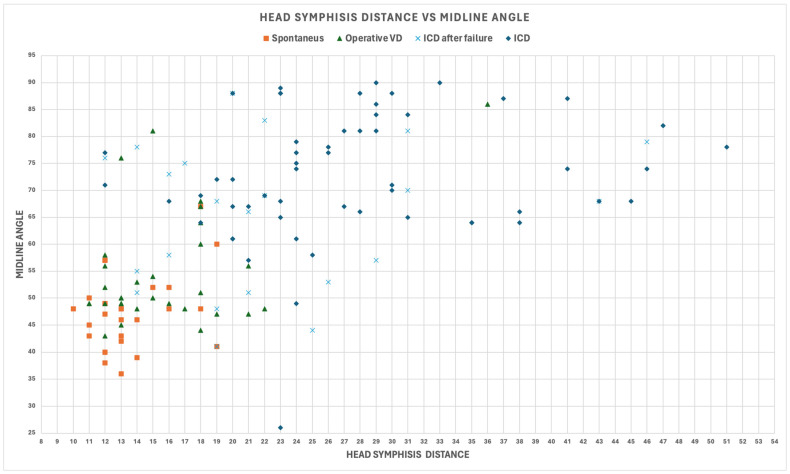
Midline angle (MLA) and fetal head–symphysis distance (HSD) for all patients involved in the study.

The outcomes of delivery were the following: 56 intrapartum cesarean deliveries (ICDs), 22 intrapartum cesarean deliveries after failure (ICDs after failure), 31 operative vaginal deliveries (OVDs), and 26 spontaneous deliveries (Figure 1), highlighting the moderate degree of Pearson’s correlation between the MLA and HSD due to a visible discrete correlation for spontaneous deliveries, a poor degree of correlation for all 78 ICDs, and also a large region of the plane in which there were only ICDs.

### 3.2. Findings from the AIDA’s Second Step: Machine Learning Analysis

The best performer was MLP, with the following outcomes: accuracy: 0.97, AUC: 0.993, and precision: 0.9625, followed by random forest, with the following outcomes: accuracy: 0.933, AUC: 0.97, and precision: 0.9615, and SVM, with the following outcomes: accuracy: 0.933, AUC: 0.98, and precision: 0.9157. The results for XGBoost (accuracy: 0.896, AUC: 0.96, precision: 0.9358), logistic regression (accuracy: 0.844, AUC: 0.92, precision: 0.8718), and decision tree (accuracy: 0.837, AUC: 0.84, precision: 0.8462) were less exciting. Other performance parameters for each algorithm are shown in Table 2.

**Figure 2:**
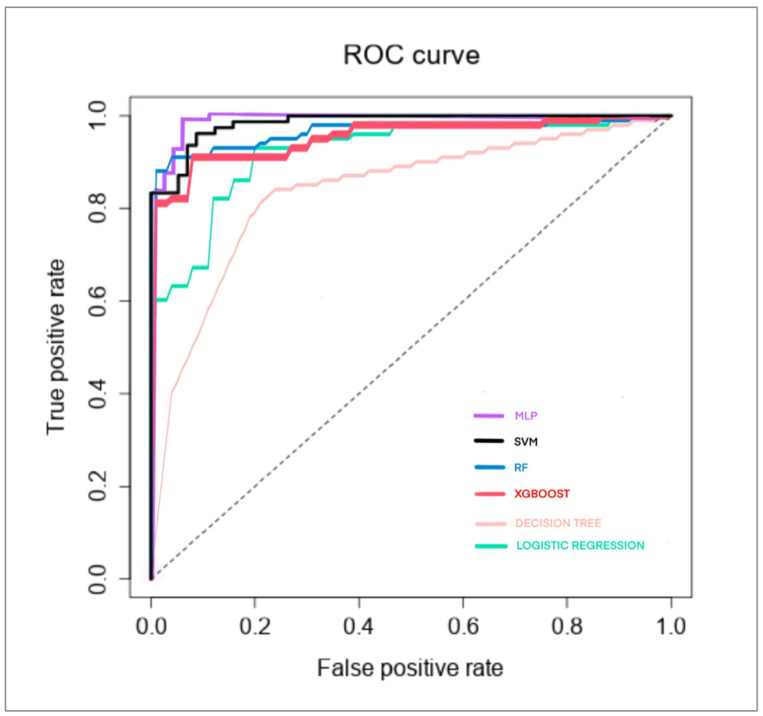
The ROC curve of six machine learning algorithms in the delivery outcome classification of our dataset.

The feature importance of the four geometric parameters, the variables in the machine learning algorithms, revealed a different hierarchy for each algorithm: (a) MLP, considered in order of importance, was AoP, HSD, MLA, and AD; (b) random forest, considered in order of importance, was MLA, HSD, AD, and AoP; (c) SVM, considered in order of importance, was HSD, AD, MLA, and AoP; (d) XGBoost, considered in order of importance, was HSD, AD, MLA, and AoP was not shown; (e) logistic regression, considered in order of importance, was HSD, MLA, AoP, and AD; (f) decision tree, considered in order of importance, was MLA, AoP, AD, and HSD. The ROC curve for each algorithm are shown in Figure 2.

### 3.3. Findings from the AIDA’s Third Step: AIDA Classes

The results of the decision tree (DT) algorithm applied to each pair of geometric parameters, shown in Table 3, returned the following cut-off values: (a) pair AoP and AD, ICD for AoP >= 144.5° or AoP < 101.5° and ICD for AD >= 67; (b) pair HSD and AD, ICD for HSD >= 19.5 and ICD for AD >= 70.5; (c) pair MLA and AD, ICD for AD >= 65.5; (d) pair HSD and AoP, ICD for HSD >= 19.5; (e) pair AoP and MLA, ICD for MLA >= 60.5; (f) pair HSD and MLA, ICD for HSD >= 19.5 and ICD for MLA >= 62. 

For the angle of progression, we obtained two cut-off values, indicating the presence of ICD for both AoP < 101.5° and AoP >= 144.5°. This means that in our dataset, the delivery outcome was ICD for the values of AoP from 72 (min value) to <101.5°, ICD for the values of AoP from 144.5° to 192° (max value), and non-ICD for the values of AoP from 101.5° to 144.5°. In our dataset, we color-coded each AoP value <101.5° and those >=144.5° in red, and those between 101.5° and 144.5° in green.

For the fetal head–symphysis distance, we obtained a cut-off value indicating the presence of ICD if HSD >= 19.5. This means that in our dataset, the delivery outcome was ICD for the values of HSD between 19.5 and 51 (max value) and non-ICD for the values of HSD between 10 (min value) and 19.5. In our dataset, we color-coded each HSD value >= 19.5 in red and those between 10 and 19.5 in green.

For the midline angle, we obtained two cut-off values, indicating the presence of ICD if MLA > 62° (DT between HSD and MLA) and MLA > 60.5° (DT between AoP and MLA). This means that in our dataset, the delivery outcome was ICD for the values of MLA between 62° and 90° (max value in our dataset) and non-ICD for values of MLA between 26° (min value in our dataset) and 60.5°. In our dataset, we color-coded each MLA value >= 62° in red and those between 26° and 60.5° in green. The values from 60.5° to 62° were considered a range of cut-off values and were colored yellow. 

For the asynclitism degree, we obtained three cut-off values, indicating the presence of ICD if AD > 65.5 mm (DT between MLA and AD), AD > 67 mm (DT between AoP and AD), and AD > 70.5 mm (DT between HSD and AD). This means that in our dataset, the delivery outcome was ICD for the values of AD from 70.5 mm to 95 mm (max value in our dataset) and non-ICD for the values of AD from 4 mm (min value in our dataset) to 65.5 mm. In our dataset, we color-coded each AD value >= 70.5 mm in red and those between 4 mm and 65.5 mm in green. The values from 65.5 mm to 70.5 mm were considered a range of cut-off values and were colored yellow.

A total of 135 patients involved in the study were classified into the defined five AIDA classes depending on the contribution of every geometric parameter to the delivery outcome: red (ICD), green (non-ICD), or yellow (high probability of ICD): (a) AIDA class 4, with all four parameters in the red or yellow zone. In our dataset, the delivery outcome was twenty ICDs and three ICDs after failure; (b) AIDA class 3, with three parameters in the red or yellow zone and one parameter in the green zone. In our dataset, the delivery outcome was twenty-six ICDs, nine ICDs after failure, and three operative VDs; (c) AIDA class 2, with two parameters in the red or yellow zone and two parameters in the green zone. In our dataset, the delivery outcome was nine ICDs, seven ICDs after failure, and four operative VDs; (d) AIDA class 1, with one parameter in the red or yellow zone and three parameters in the green zone. In our dataset, the delivery outcome was one ICD, three ICDs after failure, six operative VDs, and four spontaneous deliveries: (e) AIDA class 0, with zero parameters in the red or yellow zone and four parameters in the green zone. In our dataset, the delivery outcome was zero ICDs, zero ICDs after failure, eighteen operative VDs, and twenty-two spontaneous deliveries.

### 3.4. Findings from the AIDA’s Fourth Step: Reliability of the Predictions

Five different random samples were generated using the 70–30 percentage split and the random seed, an integer value between 1 and 1000. Altering this seed changed the assignment of individual rows of data to each sample. The five different values used for the random seed were 1, 0, 250, 500, and 750. The five 95-record samples were used to make the predictions on the respective 40 records and were used for the three following algorithms: RF, SVM, and MLP. The performance is reported in Table 4.

Two algorithms, RF and MLP, were the same confusion matrix and performance in each of the five samples, ranging from the maximum in seed1 (accuracy: 0.95, AUC: 0.992, precision: 1.0, specificity: 1.0, recall: 0.92, F1 score: 0.9583) and the minimum in seed250 (accuracy: 0.7750, AUC: 0.9115, precision: 0.6522, specificity: 0.6667, recall: 0.9375, F1 score = 0.7692). The SVM in seed1 had the same confusion matrix and performance of the other two algorithms, with a minimum in seed250 (accuracy: 0.7500, AUC: 0.9089, precision: 0.6250, specificity: 0.6250, recall: 0.9375, F1 score: 0.7500). The different performance of the algorithms confirms different record content between the samples that are in the estimation and those in the validation samples. The five different 70–30 separation samples were composed of five groups of 40 predictions, a total of 200 predictions, of which 111 were from distinct patients and 89 repetitions, as some patients were selected multiple times using the seeds tool. All of the 200 forecasts were then grouped according to the AIDA class.

The confusion matrix and corresponding metrics of the three machine learning algorithms in the delivery outcome prediction for every AIDA class, where a positive value represents an ICD delivery outcome and a negative value denotes non-ICD, are reported in Table 5, which are as follows:

(a) AIDA class 0, which includes all patients who had all four green parameters. The better performers were both RF and MLP, as the accuracy was 1.0, the NPV was 1.0, and the specificity was 1.0;
(b) AIDA class 4, which includes all patients who had all four red or yellow parameters. The better performers were both RF and SVM, as the accuracy was 1.0, the PPV was 1.0, the recall was 1.0, and the F1 score was 1;
(c) AIDA class 3, which includes all patients who had three red or yellow parameters and one green parameter. The better performer was RF, as the accuracy was 0.92, the PPV was 0.9167, the NPV was 1.0, the recall was 1.0, the specificity was 0.3333, and the F1 score was 0.9565;
(d) AIDA class 2, which includes all patients who had two red or yellow parameters and two green parameters. The better performer was RF, as the accuracy was 0.6111, the PPV was 0.6176, the NPV was 0.5, the recall was 0.9545, the specificity was 0.0714, and the F1 score was 0.75;
(e) AIDA class 1, which includes all patients who had one red or yellow parameter and three green parameters. The better performer was MLP, as the accuracy was 0.6667, the PPV was 0.2, the NPV was 0.9, the recall was 0.5, the specificity was 0.6923, and the F1 score was 0.2857.

## 4. Discussion

The progress of labor can be monitored not only using traditional obstetric practices but also using intrapartum ultrasounds [11]. In fact, in the second stage of labor, there are ultrasound parameters that allow you to monitor the progress of the fetus’s descent into the birth canal and may predict the labor outcome: spontaneous, whether operative VD should be attempted, or if ICD is preferable or necessary [12]. An ultrasound examination should include more just than the assessment of the fetal station because rotational movements are necessary for the fetus to descend. These rotational movements of the fetal head and shoulders are often called the cardinal movements [13]. The fetal position and rotation can be examined with an ultrasound: the four recorded parameters that we analyzed, namely the midline angle (MLA), angle of progression (AoP), fetal head–symphysis distance (HSD), and asynclitism Degree (AD), are the most reported in the literature [12,14].

**Figure 3:**
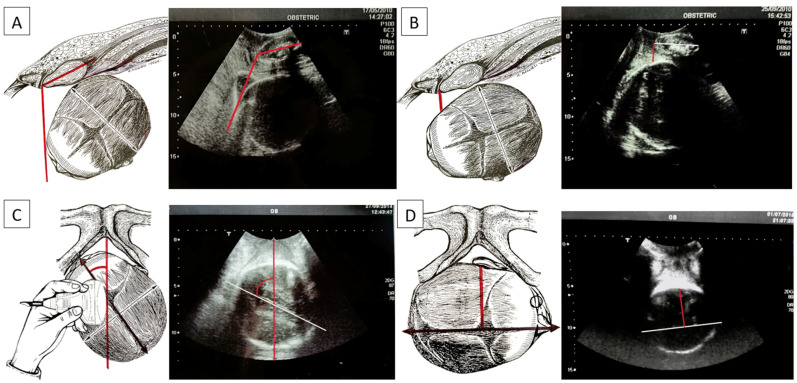
(**A**) Angle of progression (AoP): the drawing on the right and the US photo on the left show the AoP (red line) with the fetal head in the occiput anterior position; (**B**) fetal head–symphysis distance (HSD): the drawing on the right and the US photo on the left show the HSD (red line) with the fetal head in the occiput anterior position; (**C**) midline angle (MLA): the drawing on the right and the US photo on the left shows the MLA with the fetal head in the left occiput posterior position (white/black line: midline, the echogenic line between the two cerebral hemispheres; red line: anteroposterior diameter of the pubis). Longitudinal translabial sonography detected the MLA; (**D**) asynclitism degree (AD): the drawing on the right and the US photo on the left show the AD with the fetal head in the right occiput posterior position with anterior asynclitism (white/black line: midline, the echogenic line between the two cerebral hemispheres; red line: anterior asynclitism degree, perpendicular to the white/black line).

The AoP is the angle identified using a line passing through the central part of the pubic symphysis and another tangent to the fetal skull via longitudinal scanning (Figure 3A). Using a geometric model, Barbera et al. [14] developed an algorithm to assign a specific set of theoretical intervals of angles at the mid-point of a line drawn between the ischial spines (at zero station: lower angle: 96°, mean angle: 99°, upper angle: 102°) and at the other intervals of angles at the other clinical stations (−5 to +5). This measurement, initially termed the “angle of descent”, is now scientifically referred to as the angle of progression (AoP). Upon reaching full dilatation in all women, an AoP greater than 120° was consistently associated with head engagement, as determined via clinical examination. Notably, in all instances of vaginal delivery, there was a uniform increase in the AoP, and spontaneous delivery occurred in 90% when the AoP exceeded 120° [15]. 

Skinner et al. [14] reported a comparative systematic review and meta-analysis on the mean differences in the AoP, at rest and with pushing, between women having complicated OVBs and women having uncomplicated OVBs. Nassr et al. [16] reported a systematic review evaluating eight prospective cohort studies reporting on 887 pregnancies. Subgroup analyses were conducted based on angle of progression ranges from 108° to 119°, 120° to 140°, and 141° to 153°. In our database, our AIDA algorithm was found for the following AoP interval cut-off angles: ICDs for AoP < 101.5° and AoP >= 144.5° and non-ICDs for AoP >= 101.5° and AoP < 144.5°.

The HSD is evaluated via translabial sonography in a lithotomy position of the patient and on the midsagittal scan. On this view, two landmarks are identified: the maternal pubic symphysis and the fetal head. The HSD is the minimum distance between the lower margin of the maternal pubic symphysis and the fetal parietal bone (Figure 3B). If HSD > 20 mm, the head is not engaged. If HSD < 20 mm, it is engaged, having reached at least the ischial spines. For values less than 10 mm, the head is between +2 and +3, according to the ACOG classification. A low HSD value associated with other parameters (high angle of progression and midline angle <45°) depicts a good delivery outcome [17]. In our database, our algorithms were found for HSD cut-off values of 19.5 mm between non-ICDs for HSD < 19.5 mm and ICDs for HSD >= 19.5 mm.

In the MLA, the angle formed by the fetal head midline (the echogenic line between the two cerebral hemispheres) and the anteroposterior diameter of the pubis is measured, known as the “midline” angle (Figure 3C). This midline angle measurement appears to correlate with the clinically assessed station, particularly in fetuses with an occiput anterior position. If the midline angle is less than 45°, the station is typically ≥+2 cm in many cases. Conversely, in fetuses with a midline angle >45° or one that is not clearly measurable, the clinical assessment often indicates the head station as <+2 cm from the ischial spines. The midline angle value has also been shown to be helpful in predicting the success of operative vaginal delivery, as no failures were reported in fetuses with an occiput anterior position when the midline angle was ≤45°. When the MLA is less than or equal to 45°, internal rotation has occurred and the head has rotated. If it is greater than 45°, internal rotation has not occurred [18]. 

In Skinner [14], a comparative systematic review and meta-analysis on the mean differences in MLA, at rest and with pushing, between women having complicated OVBs and women having uncomplicated OVBs were reported. In our database, our AIDA algorithm identified an interval cu-off for the MLA, with a lower value of 60.5 mm and an upper value of 62.0 mm, which delineates between non-ICDs for MLA < 60.5 mm and ICDs for MLA >= 70.5 mm.

Asynclitism is evaluated via vaginal digital examination, but the AD can be detected via intrapartum ultrasound, which improves the diagnosis through a transabdominal ultrasound that diagnoses the squint sign (which is the visualization of only one fetal orbit, which is a landmark that indicates that the head is twisted in the birth canal). With translabial US, anterior asynclitism is defined when the midline shifts towards the sacrum, while posterior asynclitism is defined when the midline shifts towards the pubis. To evaluate the degree of asynclitism, the distance in millimeters between the midline and the presented parietal bone was evaluated using translabial ultrasound in the longitudinal plane (Figure 3D). A moderate degree of asynclitism is considered a normal adaptation of the fetal head in the birth canal, and it results in normal labor and spontaneous delivery, specifically when the fetal head in the occiput anterior position engages with an anterior asynclitism. High-grade asynclitism can be associated with maternal and fetal complications, the most common of which are arrest disorders of the fetal head during descent into the birth canal [19,20]. In our database, our AIDA algorithm identified an interval cut-off for AD, with a lower value of 65.5 mm and an upper value of 70.5 mm for ICDs for AD >= 70.5 mm and non-ICDs for AD < 65.5 mm.

All these ultrasonographic parameters and rotations in the fetal position are depicted in Figure 3.

In daily practice, the physician’s decision to perform a delivery via an ICD or not is also based on obstetric experience but also on scientific evidence. Moreover, it is precisely this scientific evidence that we have tried to propose a new method based on the construction of an algorithm based on the analysis of parameters with AI. The AIDA algorithm adds a further element of granularity by assigning one of the five AIDA classes to the case under consideration to better understand how the ICD prediction of a machine learning algorithm can be explained and traced back to an explanation and understanding of the case under consideration, foregrounding the two factors: causality and explainability of artificial intelligence in medicine [21].

In our retrospective analysis of patients who birthed a child with the relevant ultrasound parameters collected, including the MLA, AoP, HSD, and AD, all the patients involved in the study, as represented in Figure 3, were assigned an AIDA class. 

**Figure 4:**
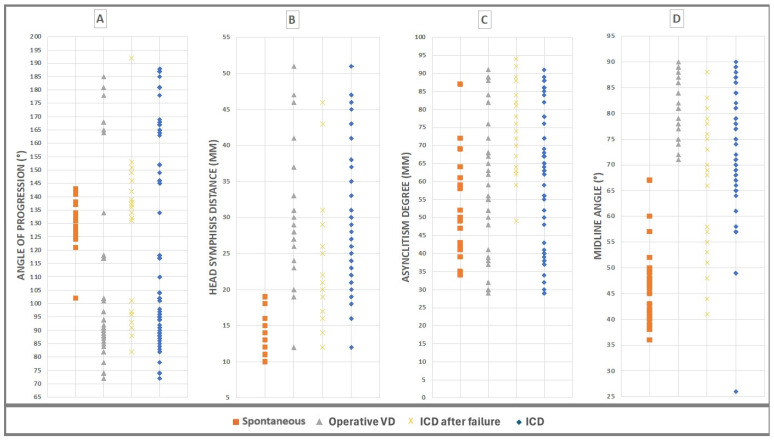
The values of the four geometric parameters, AoP, HSD, MLA, and AD, measured at the same time for the 135 cases of the patients involved in this study and grouped by delivery outcome. (**A**) Angle of progression (AoP), values between min 72° and max 192°. (**B**) Fetal head–symphysis distance (HSD), values between min 10 mm and max 51 mm. (**C**) Asynclitism degree (AD), values between min 4 mm and max 95 mm. (**D**) Midline angle (MLA): values between min 26° and max 90°.

Figure 4 shows the values of the four geometric parameters, AoP, HSD, MLA, and AD, measured at the same time for the 135 cases of the patients involved in this study and also the near impossibility of defining with just one of the four parameters which “cut-offs” should be considered in predicting the delivery outcome. Underlying the AIDA (artificial intelligence dystocia algorithm), the algorithm built is as follows: In our sample of 135 patients, we identified, using a decision tree, the cut-off values for each geometric parameter by cross-referencing the data of the four parameters in pairs of two. This process helped delineate the boundaries between the “no ICD” zone (green) and the “ICD zone” (red). In cases where different and close cut-off values were found, we defined the "may be ICD" zone (yellow).

The AIDA algorithm’s ability to classify and predict the outcome of delivery (ICD or non-ICD) was tested. Our dataset was subjected to delivery outcome classification using five machine learning algorithms: random forest, XGBoost, logistic regression, decision tree, support vector machine (SVM), and a neural network called multi-layer perceptron (MLP). The results displayed in Table 2 were quite impressive, with MLP, SVM, and RF classifying ICD (TP) in 77, 76, and 75 of the 78 patients with ICD delivery outcomes, respectively. The delivery outcome prediction of our dataset was performed using the same machine learning algorithms.

In AIDA class 4 and AIDA class 0, the three algorithms also provided almost equal predictions. AIDA class 4 highlighted a series of negative parameters for a spontaneous vaginal birth or operative VD, as the HSD and AoP did not assume an adequate value for a spontaneous delivery, therefore the MLA and AD were excessively unfavorable for the progression of the fetal head into the birth canal. All four geometric parameters had a value in the ICD zone (red) or “may be in the ICD zone” (yellow). For all three algorithms, the precision or positive predictive value (PPV) was 1.0; for the MLP, the accuracy and recall were 0.9677, and the F1 score was 0.9836; and for RF and SVM, the accuracy, recall, and F1 scores were 1.0. It is practically certain that the delivery was ICD. Of the 23 cases in AIDA class 4 in our database, as many as 18 were drawn in the 5 70/30 samples, of which 13 were present multiple times, for a total of 31 predictions for each algorithm. Out of the total 93 predictions made by the three algorithms, only the MLP was classified as a FN when classifying an ICD (ICD classified as non-ICD). 

On the contrary, the AIDA class 0 highlighted a series of positive parameters for a spontaneous vaginal birth or operative VD. All four geometric parameters took a value in the “no ICD” zone (green). For all three algorithms, the negative predictive value (NPV) was 1.0; for the SVM, the accuracy and specificity were 0.9853; and for RF and MLP, the accuracy and specificity were 1.0. These values demonstrated the absolute non-necessity of carrying out an ICD. Of the 40 cases in AIDA class 4 in our database, as many as 38 were drawn in the 5 70/30 samples, of which 30 were present multiple times, for a total of 68 predictions for each algorithm. Out of the total 204 predictions made by the three algorithms, only SVM classified a non-ICD as a FP (non-ICD classified as ICD). In AIDA class 3, three geometric parameters took a value in the ICD zone (red) or “may be the ICD zone” (yellow), and one in the no ICD zone (green).

Of the 38 cases in AIDA class 3 in our database, 35 were ICD, and 3 were non-ICD, and as many as 30 were drawn in the 5 70/30 samples, of which 20 were present multiple times, for a total of 50 predictions for each algorithm. Two algorithms provided almost equal predictions. For both RF and SVM, the TP was 44, equal to 27 delivery outcome ICDs, confirmed as multiple predictions and FNs (the delivery outcome was ICD and was classified as non-ICD), which was zero, and the recall was 1. Of the three non-ICDs (operative VDs) contained in our database, there were two presentations in the samples, and RF classified it as non-ICD (TN = 2) in one case, while it classified the other two cases as ICD (FP = 4). SVM, on the other hand, classified them all as ICD (FP = 6). 

The 20 cases in AIDA class 2 and the 14 cases in AIDA class 1 in our database were not sufficient for any definitive conclusion, but they are data to reflect on and think about in future clinical ultrasound studies.

In our study, having simultaneously measured four geometric parameters, it was possible to build an AIDA (artificial intelligence dystocia algorithm), which can be a helping decision support system in the binary prediction of ICD or non-ICD, with a high likelihood for those cases falling in AIDA class 0 and AIDA class 4, and a useful comparison for those falling in AIDA class 3. 

Further investigations could be conducted on larger data samples investigating the interference and prevalence phenomena between geometric parameters, even in the intermediate AIDA classes, i.e., results like those obtained for the two extremes AIDA classes 0 and 4. A final notation regarding the type of algorithms used: the MLP algorithm, which uses a neural network, not only provided classifications but also assigned a probability to each examined case as ICD or non-ICD and was expressed based on the prevalence of either prediction.

### Highlights from the AIDA

In the AIDA, there are four aspects to highlight: the method, the data, the training, the predictions, and their reliability.

The method is divided into the four described steps. The first step is to verify that the four geometric parameters are uncorrelated or have non-significant correlations. The second step is to choose at least three of the best-performing supervised machine algorithms to have higher reliability if at least two of the three algorithms provide the same performing outcome. The third step is to identify, in the dataset under consideration, the cut-off values of the four geometric parameters associated with the ICD delivery outcome and those associated with the non-ICD delivery outcome. The fourth step is to perform the prediction of the outcome and its reliability.

Regarding the data, there is no consensus on which intrapartum ultrasound measures are more accurate and which “cut-offs” should be considered in clinical practice [14]. The cut-off values may change as the countries and datasets are analyzed, and the use of historical clinical data may contain inaccuracies or biases. A larger dataset than the one used in this paper could allow all techniques for eliminating data containing inaccuracies or biases so that the AIDA method would be applied to data purified of biases.

Regarding the training, predictions, and reliability, the research highlighted that if all four geometric parameters are in the red or yellow zone, there is a high likelihood that the delivery outcome will be ICD (AIDA class 4). In our dataset, the physician-practiced delivery outcome and AIDA prediction agreed 100% two times, with the algorithms RF and SVM achieving accuracy, PPV, F1 score, and recall values of 1. Slightly lower agreement was observed with MLP, with an accuracy of 0.9677, PPV of 1, recall of 0.9677, and F1 score of 0.9836). On the other hand, if all four parameters are in the green zone, there is a high likelihood that the delivery outcome will be non-ICD (AIDA class 0). In our dataset, the physician-practiced delivery outcome and AIDA prediction agreed 100% two times, with the algorithms RF and MLP achieving accuracy, NPV, and specificity scores of 1. This was observed to a lesser extent with SVM (accuracy = 0.9853, NPV = 1, specificity = 0.9853). For both AIDA classes 4 and 0, the AIDA method agreed with the physician’s decisions, making their predictions reliable and explainable. If three geometric parameters are in the red or yellow zone and one is in the green zone (AIDA class 3), there is a high likelihood that the ICD physician-practiced delivery outcome and AIDA prediction agreed 100% two times using the RF and SVM algorithms (recall = 1); the differences in the predictions were regarding the delivery outcome, i.e., non-ICD for the physician and ICD for the AIDA, classified as a FP or FN. There are too few cases to be able to make considerations and draw conclusions, but with a larger dataset, it would be possible to investigate both FPs and FNs to see whether the current clinical decision-making process outperforms the AIDA or not. In our database, the total number of cases falling into these three AIDA classes was 101 out of 135. The remaining 34 cases, divided into AIDA classes 1 and 2, were not sufficient for calculations or considerations.

## 5. Conclusions

The results achieved with the AIDA are preliminary but are useful for building a larger database on which to study a large number of labors and their birth outcomes based on the four geometric parameters reported in this paper. It is a major limitation that the inclusions and outcomes vary in the papers, and no studies have a randomized design [13]. An unresolved question is whether a prediction should influence clinical management [12]. The AIDA could be a method to investigate whether and how we could arrive at a robust ground truth or gold standard. 

## Figures and Tables

**Table 1:** Pearson’s correlations for all 135 patients involved in the study.

Pearson’s Correlation		*PC*		*p*	Note
Apgar score at 1 min	Apgar score at 5 min	0.8	Very strong	8.42 × 10^−32^	***
Angle of progression	Asynclitism degree	0.36	Weak	0.00002	***
Head–symphysis distance	Asynclitism degree	0.18	Very weak	0.03	*
Midline angle	Asynclitism degree	0.14	Very weak	0.09	
Angle of progression	Head–symphysis distance	−0.12	Very weak	0.14	
Angle of progression	Midline angle	−0.27	Weak	0.001	***
Head–symphysis distance	Midline angle	0.55	Moderate	8 × 10^−12^	***
Apgar score at 1 min	Angle of progression	0.19	Very weak	0.02	*
Apgar score at 1 min	Head–symphysis distance	−0.12	Very weak	0.16	
Apgar score at 1 min	Midline angle	−0.31	Weak	0.0002	***
Apgar score at 1 min	Asynclitism degree	−0.2	Weak	0.02	*
Apgar score at 5 min	Angle of progression	0.18	Very weak	0.03	*
Apgar score at 5 min	Head–symphysis distance	−0.15	Very weak	0.07	
Apgar score at 5 min	Midline angle	−0.24	Weak	0.004	**
Apgar score at 5 min	Asynclitism degree	−0.19	Very weak	0.02	*

* *p* < 0.05; ** *p* < 0.01; *** *p* < 0.001.

**Table 2:** The performance of six machine learning algorithms in the delivery outcome classification of our dataset.

Algorithm	Accuracy	AUC	PPV	NPV	Recall	Specificity	F1 Score	TP	FP	FN	TN
MLP	0.970	0.993	0.9625	0.9818	0.9872	0.9474	0.9747	77	3	1	54
Random forest	0.933	0.97	0.9615	0.8947	0.9259	0.9444	0.9433	75	3	6	51
SVM	0.933	0.98	0.9157	0.9615	0.9744	0.8772	0.9441	76	7	2	50
XGBoost	0.896	0.96	0.9358	0.8421	0.8902	0.9057	0.9125	73	5	9	48
Logistic regression	0.844	0.92	0.8718	0.8070	0.8608	0.8214	0.8662	68	10	11	46
Decision tree	0.837	0.84	0.8462	0.8246	0.8684	0.7966	0.8571	66	12	10	47

**Table 3:** Cut-off values for ICD and non-ICD for each geometric parameter.

	AoP (°)	AD (mm)	HSD (mm)	MLA (°)
AoP–AD	<101.5	>=67		
	>=144.5			
HSD–AD		>=70.5	>=19.5	
MLA–AD		>=65.5		
HSD–AoP			>=19.5	
AoP–MLA				>=60.5
HSD–MLA				>=62.0

**Table 4:** The performance of three machine learning algorithms, RF, SVM, and MLP, for each sample with the corresponding seed identifier number.

Random Seed	Algorithm	AUC	Accuracy	PPV	NPV	Recall	Specificity	F1 Score	TP	FP	FN	TN
Seed 1	RF	0.9920	0.9500	1.0000	0.8824	0.9200	1.0000	0.9583	23	0	2	15
	SVM	0.9920	0.9500	1.0000	0.8824	0.9200	1.0000	0.9583	23	0	2	15
	MLP	0.9920	0.9500	1.0000	0.8824	0.9200	1.0000	0.9583	23	0	2	15
Seed 0	RF	0.9733	0.9500	0.9259	1.0000	1.0000	0.8667	0.9615	25	2	0	13
	SVM	0.9493	0.9000	0.8621	1.0000	1.0000	0.7333	0.9259	25	4	0	11
	MLP	0.9733	0.9500	0.9259	1.0000	1.0000	0.8667	0.9615	25	2	0	13
Seed 250	RF	0.9115	0.7750	0.6522	0.9412	0.9375	0.6667	0.7692	15	8	1	16
	SVM	0.9089	0.7500	0.6250	0.9375	0.9375	0.6250	0.7500	15	9	1	15
	MLP	0.9115	0.7750	0.6522	0.9412	0.9375	0.6667	0.7692	15	8	1	16
Seed 500	RF	0.9275	0.8250	0.7407	1.0000	1.0000	0.6500	0.8511	20	7	0	13
	SVM	0.9200	0.8250	0.7407	1.0000	1.0000	0.6500	0.8511	20	7	0	13
	MLP	0.9275	0.8250	0.7407	1.0000	1.0000	0.6500	0.8511	20	7	0	13
Seed 750	RF	0.9886	0.8750	0.7222	1.0000	1.0000	0.8148	0.8387	13	5	0	22
	SVM	0.9858	0.8500	0.6842	1.0000	1.0000	0.7778	0.8125	13	6	0	21
	MLP	0.9886	0.8750	0.7222	1.0000	1.0000	0.8148	0.8387	13	5	0	22

**Table 5:** Confusion matrix and performance of RF, SVM, and MLP in the delivery outcome prediction on all 200 predictions grouped according to AIDA class.

AIDA Class	Algorithm	Accuracy	PPV	NPV	Recall	Specificity	F1 Score	TP	FP	FN	TN
Class 0	RF	1	NA	1	NA	1	NA	0	0	0	68
	SVM	0.9853	0.00	1	NA	0.9853	NA	0	1	0	67
	MLP	1	NA	1	NA	1	NA	0	0	0	68
Class 1	RF	0.5333	0.00	0.80	0.00	0.6154	NA	0	5	2	8
	SVM	0.5333	0.00	0.80	0.00	0.6154	NA	0	5	2	8
	MLP	0.6667	0.20	0.90	0.50	0.6923	0.2857	1	4	1	9
Class 2	RF	0.6111	0.6176	0.50	0.9545	0.0714	0.750	21	13	1	1
	SVM	0.5833	0.60	0.00	0.9545	0.00	0.7368	21	14	1	0
	MLP	0.5833	0.60	0.00	0.9545	0.00	0.7368	21	14	1	0
Class 3	RF	0.920	0.9167	1	1	0.3333	0.9565	44	4	0	2
	SVM	0.880	0.880	NA	1	0.00	0.9362	44	6	0	0
	MLP	0.780	0.8667	0.00	0.8864	0.00	0.8764	39	6	5	0
Class 4	RF	1	1	NA	1	NA	1	31	0	0	0
	SVM	1	1	NA	1	NA	1	31	0	0	0
	MLP	0.9677	1	0.00	0.9677	NA	0.9836	30	0	1	0

## Data Availability

The authors of the study are custodians of the data in anonymous form, which can possibly be provided to anyone who makes a reasonable request.

## References

[1] Nizard J., Haberman S., Paltieli Y., Gonen R., Ohel G., Le Bourthe Y., Ville Y. (2009). Determination of fetal head station and position during labor: A new technique that combines ultrasound and a position-tracking system. Am. J. Obstet. Gynecol..

[2] Hung C.M.W., Chan V.Y.T., Ghi T., Lau W. (2021). Asynclitism in the second stage of labor: Prevalence, associations, and outcome. Am. J. Obstet. Gynecol. MFM.

[3] (2015). ACOG Practice Bulletin No. 154: Operative Vaginal Delivery. Obstet. Gynecol..

[4] Dupuis O., Silveira R., Zentner A., Dittmar A., Gaucherand P., Cucherat M., Redarce T., Rudigoz R.C. (2005). Birth simulator: Reliability of transvaginal assessment of fetal head station as defined by the American College of Obstetricians and Gynecologists classification. Am. J. Obstet. Gynecol..

[5] Sherer D.M., Abulafia O. (2003). Intrapartum assessment of fetal head engagement: Comparison between transvaginal digital and transabdominal ultrasound determinations. Ultrasound Obstet. Gynecol..

[6] Ben-Haroush A., Melamed N., Kaplan B., Yogev Y. (2007). Predictors of failed operative vaginal delivery: A single-center experience. Am. J. Obstet. Gynecol..

[7] Rizzo G., Ghi T., Henrich W., Tutschek B., Kamel R., Lees C.C., Mappa I., Kovalenko M., Lau W., Eggebo T. (2022). Ultrasound in labor: Clinical practice guideline and recommendation by the WAPM-World Association of Perinatal Medicine and the PMF-Perinatal Medicine Foundation. J. Perinat. Med..

[8] Beck R., Malvasi A., Kuczkowski K.M., Marinellim E., Zaami S. (2019). Intrapartum sonography of fetal head in second stage of labor with neuraxial analgesia: A literature review and possible medicolegal aftermath. Eur. Rev. Med. Pharmacol. Sci..

[9] (2020). Operative Vaginal Birth: ACOG Practice Bulletin, Number 219. Obstet Gynecol..

[10] Malgieri L., Cinnella G., Beck R., Malvasi A. (2023). Ontologies, Machine Learning and Deep Learning in Obstetrics. Practical Guide to Simulation in Delivery Room Emergencies.

[11] Ghi T., Eggebø T., Lees C., Kalache K., Rozenberg P., Youssef A., Salomon L.J., Tutschek B. (2018). ISUOG Practice Guidelines: Intrapartum ultrasound. Ultrasound Obstet. Gynecol..

[12] Usman S., Hanidu A., Kovalenko M., Hassan W.A., Lees C. (2023). The sonopartogram. Am. J. Obstet. Gynecol..

[13] Eggebø T.M., Hjartardottir H. (2024). Descent of the presenting part assessed with ultrasound. Am. J. Obstet. Gynecol..

[14] Skinner S.M., Giles-Clark H.J., Higgins C., Mol B.W., Rolnik D.L. (2023). Prognostic accuracy of ultrasound measures of fetal head descent to predict outcome of operative vaginal birth: A comparative systematic review and meta-analysis. Am. J. Obstet. Gynecol..

[15] Barbera A.F., Imani F., Becker T., Lezotte D.C., Hobbins J.C. (2009). Anatomic relationship between the pubic symphysis and ischial spines and its clinical significance in the assessment of fetal head engagement and station during labor. Ultrasound Obstet. Gynecol..

[16] Nassr A.A., Berghella V., Hessami K., Bibbo C., Bellussi F., Robinson J.N., Marsoosi V., Tabrizi R., Safari-Faramani R., Tolcher M.C. (2022). Intrapartum ultrasound measurement of angle of progression at the onset of the second stage of labor for prediction of spontaneous vaginal delivery in term singleton pregnancies: A systematic review and meta-analysis. Am. J. Obstet. Gynecol..

[17] Youssef A., Maroni E., Cariello L., Bellussi F., Montaguti E., Salsi G., Morselli-Labate A.M., Paccapelo A., Rizzo N., Pilu G. (2014). Fetal head-symphysis distance and mode of delivery in the second stage of labor. Acta Obstet. Gynecol. Scand..

[18] Ghi T., Youssef A., Maroni E., Arcangeli T., De Musso F., Bellussi F., Nanni M., Giorgetta F., Morselli-Labate A.M., Iammarino M.T. (2013). Intrapartum transperineal ultrasound assessment of fetal head progression in active second stage of labor and mode of delivery. Ultrasound Obstet. Gynecol..

[19] Malvasi A., Stark M., Ghi T., Farine D., Guido M., Tinelli A. (2012). Intrapartum sonography for fetal head asynclitism and transverse position: Sonographic signs and comparison of diagnostic performance between transvaginal and digital examination. J. Matern. Fetal Neonatal Med..

[20] Malvasi A., Vinciguerra M., Lamanna B., Cascardi E., Damiani G.R., Muzzupapa G., Kosmas I., Beck R., Falagario M., Vimercati A. (2022). Asynclitism and Its Ultrasonographic Rediscovery in Labor Room to Date: A Systematic Review. Diagnostics.

[21] Holzinger A., Langs G., Denk H., Zatloukal K., Müller H. (2019). Causability and explainability of artificial intelligence in medicine. Wiley Interdiscip. Rev. Data Min. Knowl. Discov..

